# Low-Temperature Magnetotransport of Mixed Polycrystalline Rutile, Anatase and Brookite Phases of TiO_2_

**DOI:** 10.3390/ma19132889

**Published:** 2026-07-06

**Authors:** Josipa Šćurla, Trpimir Ivšić, Gaurav Pransu, Nikola Jakupec, Neven Barišić, László Forró, Ante Bilušić

**Affiliations:** 1 Faculty of Science, University of Split, Ruđera Boškovića 33, 21000 Split, Croatia; josipa@pmfst.hr; 2 Doctoral Study of Biophysics, Faculty of Science, University of Split, 21000 Split, Croatia; 3 Division of Physical Chemistry, Ruđer Bošković Institute, 10000 Zagreb, Croatia; tivsic1@irb.hr (T.I.);; 4 Laboratory of Physics of Complex Matter, Ecole Polytechnique Fédérale de Lausanne, CH-1015 Lausanne, Switzerland; 5 Institute of Physics, Bijenička Cesta 46, 10000 Zagreb, Croatia; 6 Institute of Solid State Physics, TU Wien, Wiedner Hauptstraße 8-10/E138, 1040 Vienna, Austria; 7 Department of Physics, Faculty of Science, University of Zagreb, 10000 Zagreb, Croatia; 8 Stavropoulos Center for Complex Quantum Matter, University of Notre Dame, Notre Dame, IN 46556, USA

**Keywords:** titanium dioxide (TiO_2_), anatase–rutile–brookite mixed-phase systems, magnetic susceptibility, magnetotransport

## Abstract

The anatase and rutile phases of titanium dioxide (TiO_2_) have been widely studied for their photocatalytic and electronic properties, while the brookite phase is rarely explored. The investigations of intrinsic magnetotransport in mixed-phase systems are limited. Magnetic susceptibility and magnetotransport measurements were performed between 2 K and 300 K in magnetic fields up to 12 T. Both samples with different anatase–rutile–brookite ratios exhibit Curie–Weiss susceptibility. Transport measurements reveal an insulating behavior at low temperatures with activation energies 1–20 meV and signatures of variable-range hopping (VRH) conduction. Strong positive magnetoresistance at low temperatures obeys the Efros–Shklovskii VRH model.

## 1. Introduction

Titanium dioxide TiO_2_ is a widely known transition metal oxide wide-band-gap semiconductor that has recently been investigated mostly in forms of nanoparticles, nanorods and thin films. The main applications of the material are in photovoltaic (as an electron transport layer) and photocatalytic (as an active catalyst) purposes [1,2,3,4]. Most researched crystallographic phases are anatase and rutile [5], while the brookite phase is rarely explored and electric transport measurements on this phase are lacking [6]. Anatase and rutile structures renewed scientific attention due to their remarkable performance in water photolysis and their potential for efficient charge separation and migration [7]. Brookite structure is less stable than the other two phases and electric and magnetic transport measurements on brookite single-phase samples have yet to be performed. A combination of anatase and rutile has been shown to outperform single-phase materials in photocatalytic efficiency, although the physical origin of this enhancement remains unclear [8]. Systematic experimental investigations of transport mechanisms in polycrystalline mixed-phase anatase–rutile–brookite systems remain scarce, with most studies focusing on photoinduced charge separation rather than intrinsic electronic transport [9,10]. In contrast, electrical transport in single crystals of anatase and rutile has been extensively studied, particularly in relation to oxygen vacancy concentration [11,12,13]. Magnetotransport measurements remain lacking in the literature. All polymorphs are generally considered to exhibit polaronic conduction, where a polaron is a charge carrier coupled to a local lattice distortion [14]. Weakly localized (large polaron-like) carriers are characteristic for anatase and strongly localized small polarons are found in rutile [15], while theoretical simulations for brookite predict the formation of stable small polarons [16]. Furthermore, static magnetic measurements on rutile have yielded conflicting results [17,18,19], while studies of anatase magnetism remain limited, due to difficulties in synthesizing large, high-quality crystals and their sensitivity to defect concentration.

In this work, we investigate magnetic susceptibility and magnetotransport in polycrystalline mixed-phase anatase–rutile–brookite TiO_2_ to elucidate the relationship between charge carrier localization and transport mechanisms. By combining magnetic and electrical measurements over a wide temperature and magnetic-field range, we aim to identify the dominant conduction mechanisms and their connection to defect-induced states. We show that charge transport is governed by localized carriers and variable-range hopping, with Efros–Shklovskii and Mott behavior dominating at low temperature. We also show that microstructure and phase composition may play a key role in determining the observed magnetic and transport properties. These findings provide insight into defect-driven transport in mixed-phase TiO_2_ systems. They also contribute to the broader understanding of charge transport in oxide semiconductors. To our knowledge, this is the first contribution of magnetotransport in TiO_2_ mixed-phase polymorphs.

## 2. Materials and Methods

TiO_2_ mixed-phase crystals used in this study are collected as a by-product along the synthesis of single-phase crystals. The crystals were grown by chemical vapor transport in a two-zone furnace, following a modified procedure from [20]. Commercial TiO_2_ powders (≥99.9%, anatase and rutile) with trace-metal basis purity and analytical-grade ammonium chloride (NH_4_Cl), purchased from Sigma Aldrich, were used as starting materials. The precursors were vacuum sealed in an evacuated quartz ampoule, together with NH_4_Cl as a transport agent. The source and growth zones were slowly heated to 900 °C and 725 °C, respectively, and maintained at those temperatures for 14 days. In addition to the red rutile crystals, black crystals were collected near the cold end of the ampoule and were identified as mixed-phase TiO_2_. In general, the electrical conductivity and defect structure of TiO_2_ strongly depend on synthesis conditions. Recently, in the field, extensive efforts have been devoted to defect engineering through hydrogenation, plasma treatment, UV-assisted deposition, and oxygen-deficient growth techniques. These approaches enable controlled formation of oxygen vacancies and Ti^3+^ states, thereby strongly modifying charge transport, carrier localization, and polaronic behavior [21,22,23,24]. In the present work, however, the focus is placed on the more classical chemical vapor transport (CVT) synthesis route, which provides high-quality crystalline material suitable for investigating intrinsic transport and magnetotransport properties.

Morphological and compositional characterization were performed using scanning electron microscopy (SEM) and energy-dispersive X-ray spectroscopy (EDS) with a JEOL JSM-7610F Plus microscope (JEOL Ltd., Tokyo, Japan) equipped with an Oxford Ultim Max 65 SDD X-ray analyzer. EDS mapping of the samples was conducted at accelerating voltages of up to 15 keV. The obtained spectra were analyzed to determine elemental composition and identify possible impurity contributions.

X-ray data for single crystals were recorded by ω-scans on a Rigaku Oxford Diffraction Synergy S diffractometer (Rigaku Innovative Technologies Europe s.r.o, Dolní Břežany, Czechia) with a four-circle goniometer which allows diffraction measurements over nearly all accessible diffraction angles. At low diffraction angles, an exposure time of 0.5 s per frame was used, whereas at higher diffraction angles the exposure time was increased to 60 s per frame (48 frames in total). This approach was adopted to ensure that the intensity-to-noise ratio, I/σ(I), exceeded 20 for the recorded reflections, relative to the background. Such a strategy is chosen because reflection intensities generally decrease with increasing diffraction angle.

The measurements were performed using Cu Kα radiation at 293 K. Both samples were measured using a strategy for triclinic systems (lattice: P, Laue group: 1), so as to collect the full Ewald sphere or reflections and thus obtain more representative data. Peak hunting and data reduction, including the multi-scan absorption correction, was performed using the CrysAlisPRO software package (v44.85). After peak hunting, the unit cell was found by a “brute force determination” method (available in CrysAlisPRO). The initial unit cell corresponded to the highest phase fraction in the system (rutile; a = c = 4.59 Å, b = 2.95 Å, all angles = 90°), based on the number and intensity of the reflections collected. Phase fractions were determined by using multi-crystal system identification in Ewald Explorer where parameters of the other two phases (anatase; a = c = 3.78 Å, b = 9.51 Å, all angles = 90°; and brookite; a = c = 5.45 Å, b = 9.18 Å, all angles = 90°) were inputted manually in the “find custom twin/multi-crystal phase” option. The final calculated phase fractions correspond to the number of reflections of the different unit cells (phases). These analyses revealed the presence of anatase, rutile, and brookite phases in our samples, leading to the following sample nomenclature and composition:

R82–A10–B8: 82% rutile, 10% anatase, 8% brookite.

R94–A4–B2: 94% rutile, 4% anatase, 2% brookite.

For transport measurements, R82–A10–B8 was cut into a rectangular bar 270 × 350 × 1000 µm^3^ in size. The R94–A4–B2’s shape is approximately cubic, 500 × 870 × 1500 µm^3^ in size. Electrical contacts for resistivity measurements were established using 25 µm gold wire and a silver paste in a linear 4-point configuration. Resistance was measured using a DC method from the slope of the current–voltage characteristics in the Ohmic regime, and resistivity was calculated considering the sample and contact geometry. Electrotransport measurements were performed in a cryogen-free helium gas-flow system (Cryogenic Co. Ltd., London, UK) with an integrated superconducting magnet up to 12.5 T. Temperature stabilization better than 3 mK was achieved over the range 2–300 K. Magnetoresistance measurements were performed in the magnetic field range 0–10 T for R82–A10–B8 and 0–11 T for R94–A4–B2 for two orientations of magnetic field and with a fixed step of 15 mT in all measurements. Error checking is embedded in the measurement routine to ensure no overheating effects occur. The setup included Yokogawa 7651 DC Source, an Agilent 34420A nanovoltmeter, and a Keysight B2901A source/measure unit. Magnetic moment measurements were carried out using a Quantum design magnetic-properties measurement system (MPMS) at the Institute of Physics in Zagreb, Croatia.

## 3. Results

### 3.1. Structural and Compositional Characterization

**Figure 1:**
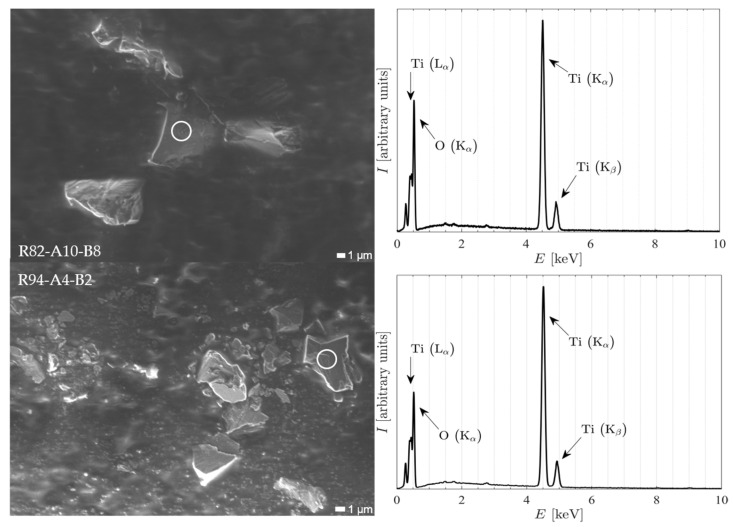
The left part of the figure shows scanning electron microscope (SEM) images, while the right part shows the energy dispersive spectra (EDS) of the studied samples. Both SEM images were taken in SEI mode, with accelerating voltage 5 keV and with ×3000 magnification. Working distance was 14.4 mm for R82-A10-B8 (upper image) and 14.6 mm for R94-A4-B2 (lower image). The EDS spectra of both samples, R82–A10–B8 (upper row) and R94–A4–B2 (lower row), are analyzed on a part of the grains indicated by a white circles on SEM images. On both EDS spectra, titanium and oxygen lines are indicated by arrows.

Figure 1 presents SEM images and EDS spectra for both samples: R82–A10–B8 is shown in the upper row, and R94–A4–B2 in the lower row. The SEM images reveal a clear polycrystalline structure in both samples, with crystallites approximately 1–10 µm in size and nanocrystallites distributed throughout the samples. For both samples, the EDS spectra were obtained from a sections of a crystallites marked by a white circles, as shown in the SEM images. Both spectra exhibit similar features: Ti and O lines are clearly visible and indicated by arrows. Additional peaks are also present, resulting either from mounting the samples with carbon tape on the SEM sample-holder stub (the line at 0.25 keV), or from handling the samples during research (the lines at 1.5 keV, 1.75 keV, and 2.7 keV and 6.4 keV correspond to aluminium, silicon, and chlorine and iron, while those at 8 keV and 9 keV are characteristic of copper). Numerical analysis of the spectra, with carbon-tape background signal excluded, shows that for R82–A10–B8 the combined signal from Ti and O accounts for 100% of the total signal measured in atomic percent (at.%), with a Ti:O ratio of 1:3.3. For R94–A4–B2, the combined Ti and O contribution is also 100%, with a Ti:O ratio of 1:2.7, as can be inferred from Table 1.

However, Ti:O ratios obtained by EDS should be interpreted with caution, as oxygen quantification by EDS is inherently unreliable. Consequently, the measured oxygen excess cannot be taken as evidence of bulk non-stoichiometry, but is more likely related to limitations of the EDS technique [25]. In this work, the elemental fraction was therefore treated only as a rough qualitative indicator of the presumed compositional trend and the underlying physical picture, rather than as a definitive measure of defect concentration.

### 3.2. Magnetic Susceptibility Measurements

**Figure 2:**
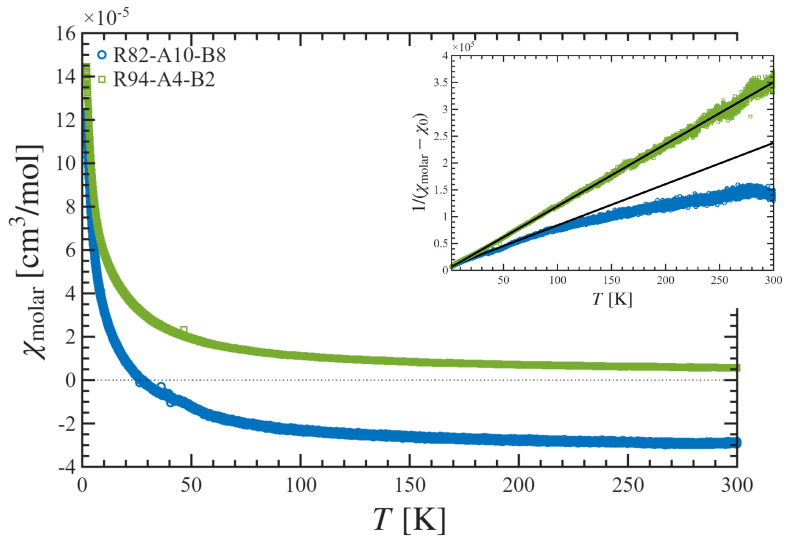
Temperature dependence of the molar magnetic susceptibility of R82–A10–B8 and R94–A4–B2. Inset shows inverse magnetic susceptibility as a function of temperature for both samples. The solid lines represent Curie–Weiss fits.

The temperature dependence of the molar magnetic susceptibility of both samples measured under a constant magnetic field of 4 T is shown in Figure 2. The magnetic field of 4 T was used to enhance the magnetic response of the as-grown, unreduced samples. In such samples, only a low concentration of charge carriers and localized magnetic moments is expected, since perfect TiO_2_ crystal is purely diamagnetic. For R82–A10–B8, the susceptibility decreases strongly in a Curie–Weiss-like manner from 1.2·10^−4^ cm^3^/mol at 2 K, and crosses zero at approximately 27 K. Above approximately 150 K, the susceptibility gradually approaches a temperature-independent value of −2.9·10^−5^ cm^3^/mol. The core diamagnetic susceptibility of TiO_2_ estimated from Pascal constants is −2.9·10^−5^ cm^3^/mol [26], implying that the high-temperature susceptibility of R82–A10–B8 arises solely from the core diamagnetism of the lattice ions. R94–A4–B2 exhibits a similar low-temperature, Curie–Weiss-like behavior, but remains paramagnetic over the entire measured temperature range. The susceptibility decreases from 1.45·10^−4^ cm^3^/mol at 2 K up to around 60 K, followed by a gradual saturation to a nearly temperature-independent value of 5.8·10^−6^ cm^3^/mol at 300 K.

The total molar susceptibility $\chi_{m}\left(T\right)$ was analyzed using a model consisting of a temperature-independent term $\chi_{0}$, attributed to the lattice and neutral defects and a temperature-dependent paramagnetic term described by the Curie–Weiss law. The inverse susceptibility was therefore fitted using the Curie–Weiss relation [14,27]:

(1) $$\chi \left(T\right)=\chi_{0}+\frac{C}{T-\theta }$$

where C is the Curie constant and $\theta_{CW}$ is the Weiss temperature. The inset of Figure 2 shows susceptibility data and the corresponding fits for both samples in the $1/(\chi -\chi_{0})$ versus temperature format.

A good fitting window of the Equation (1) for R82–A10–B8 was found at low temperatures (5–25 K), prior to the crossover to a dominant diamagnetic background. In contrast, R94–A4–B2, which remains purely paramagnetic over a wide temperature range, was fitted between 15 K and 300 K. Both fits exhibit excellent linearity with $R^{2}=0.99$. The extracted Curie–Weiss temperatures are small and negative ($\theta_{CW1}\approx -9.1\;K$ and $\theta_{CW2}\approx -3.5\;K$), indicating weak antiferromagnetic interactions. The fitted temperature ranges, $\chi_{0}$, Curie constants and derived effective magnetic moments are summarized in Table 2.

The extracted effective moments *µ*_eff_ (0.1$\mu_{B}$ and 0.08$\mu_{B}$) are only 5% of the spin-only values expected for Ti^3+^ (1.73*μ*_B_ [14]), indicating a dilute concentration of localized magnetic moments. The temperature sweeps were performed under both zero-field-cooled (ZFC) and field-cooled (FC) conditions. The two curves show no significant splitting nor any maxima in either sample, suggesting that the observed magnetic response is intrinsic.

### 3.3. Resistivity and Magnetoresistivity

**Figure 3:**
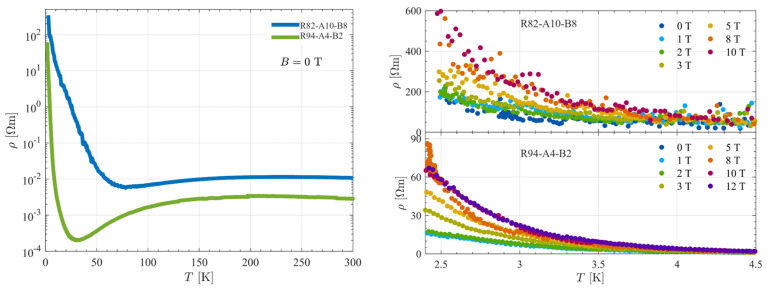
The left panel shows the temperature dependence of the resistivities for R82–A10–B8 (blue line) and R94–A4–B2 (green line) at zero magnetic field. The right panels display the low-temperature dependence of resistivities at different magnetic fields: the upper panel for R82–A10–B8, and the lower panel for R94–A4–B2.

The temperature dependence of the electrical resistivity of both samples, measured between 2 K and 300 K, is shown in the left panel of Figure 3. Two distinct temperature regimes can be identified: starting at 2 K, the resistivity decreases monotonically with increasing temperature, from 2·10^2^ Ωm for R82–A10–B8 and from 6·10^1^ Ωm for R94–A4–B2. This indicates semiconducting-like behavior up to approximately 60 K for R82–A10–B8 and up to about 20 K for R94–A4–B2. Above these temperatures, the resistivity curves exhibit metallic-like increase with temperature, extending up to around 200 K for both samples. Above 200 K, both curves become only very weakly temperature-dependent and show a very slight decrease up to 300 K. Room-temperature resistivity values are 10^−2^ Ωm and 2·10^−3^ Ωm, respectively. These values are comparable to the literature values for reduced anatase and rutile single crystals [28,29,30]. The transition between the low-temperature semiconducting regime and the higher-temperature metallic-like regime in both samples is smooth, indicating that the change in the conduction mechanism represents a crossover, rather than a phase transition. The right panels of Figure 3 display the temperature dependence of resistivity for R82–A10–B8 (upper panel) and R94–A4–B2 (lower panel) under different out-of-plane magnetic fields for temperatures between 2.5 K and 4.5 K. Both samples display positive magnetoresistance at these temperatures.

**Figure 4:**
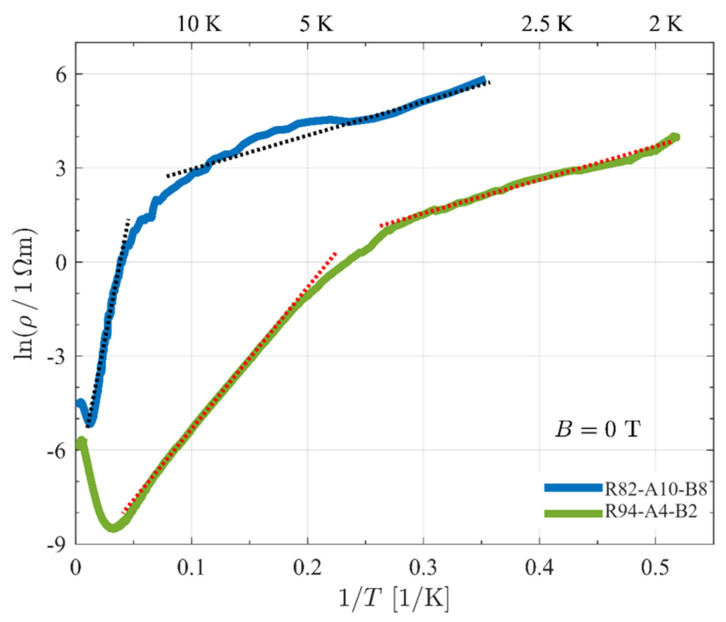
Activated representation of the resistivity data and the fits, represented by blue and red dotted lines for the low- and middle-temperature linear ranges of R82–A10–B8: 2.5–10 K (R^2^ = 0.94) and 30–55 K (R^2^ = 0.99) and R94–A4–B2: 2–3.5 K (R^2^ = 0.99) and 5–30K (R^2^ = 0.99).

When the $\rho \left(T\right)$ data are plotted in an Arrhenius representation (Figure 4), equation (2), the low (<10 K) and middle (<60 K) temperature ranges show activated behavior of the charge carriers, following the Arrhenius equation [14].

(2) $$\;\;\rho \left(T\right)=\rho_{0}e^{\frac{E_{a}}{k_{B}T}}.$$

*k*_B_ is the Boltzmann constant and *E*_a_ is the activation energy parameter extracted from the slopes of the ln(ρ) vs. 1/*T* dependence. For R82–A10–B8 (blue curve), a linear regime is observed at the lowest temperatures 2–10 K, yielding an activation energy of 0.9 meV. A second linear region with a steeper slope appears in the 30–55 K range, corresponding to *E*_a_ = 20 meV. The observed linearity in the Arrhenius representation indicates thermally activated transport, which in the low-temperature regime is consistent with nearest-neighbor hopping (NNH) between localized states [29,31,32,33]. Nevertheless, the very small low-temperature activation energy (~1 meV) suggests either the presence of very shallow states or, more likely, a different transport mechanism, such as variable-range hopping, since shallow donor levels in rutile TiO_2_ are typically located several meV below the conduction band minimum [30,34].

For R94–A4–B2 (green curve), the apparent activated behavior is observed in a narrow temperature interval 2–3.5 K, yielding an activation energy of 0.9 meV, similar to R82–A10–B8. However, due to the limited temperature range, the linearity cannot be considered conclusive, and the extracted activation energy should be treated with caution. This may indicate the presence of a different transport mechanism or the absence of a well-defined activated regime in this temperature interval. The second temperature range with the linearity in the Arrhenius plot is found between 5 K and 30 K, with the $E_{a}$ = 4 meV. All fitting ranges were confirmed with a sliding window scan and reliable $R^{2}$ values.

In addition, ρ(T) measurements performed in several constant out-of-plane magnetic fields up to 10 T for R82–A10–B8 and 12 T for R94–A4–B2 reveal an increase in resistivity, with increasing magnetic field at low temperatures (see right panels of Figure 3). Activation energies extracted from these data all show a linear increase with the magnetic field B. For R82–A10–B8 in the 2 K to 10 K range, the increase is dE_a_/dB = (0.03 ± 0.01) meV/T, and in the 30 K to 50 K range the increase is dE_a_/dB = (0.02 ± 0.02) meV/T. For R94–A4–B2, it is in the range 2–3.5 K, dE_a_/dB = (0.04 ± 0.01) meV/T, and in the 5–30 K range, dE_a_/dB = (0.03 ± 0.01) meV/T.

Besides the activated behavior of the charge carriers, there is a possibility of variable-range hopping (VRH) conduction mechanism being present at low temperatures in polaronic materials such as TiO_2_ [33,35]. Mott VRH describes transport in the systems with localized electrons which cannot move through the conduction band, having constant density of states near the Fermi level. Instead of conduction by hopping onto the closest neighbor, as in the activated (NNH) regime described above, the electron chooses an optimal hop path with respect to the distance of the hop and energy needed. This is reflected in resistivity through the following equation [36].

(3) $$\rho \left(T\right)=\rho_{0}\;exp\left[{\left(\frac{T_{0}}{T}\right)}^{p}\right]$$

where *p* = 1/4 for 3D systems and *p* = 1/3 for 2D systems. T_0_ is the parameter determined by the density of states at the Fermi level and the Mott localization length, which characterizes the exponential decay of the charge-wave function. If Coulomb interactions between hopping sites are non-negligible compared to the thermal energy k_B_T, a Coulomb gap opens in the density of states. This usually occurs at temperatures lower than those where Mott VRH is active [36]. In such cases, Mott VRH conduction transitions to Efros–Shklovskii VRH (ES-VRH), with the corresponding exponent *p* = 1/2 in the resistivity Equation (3) [37]. In this case, T_ES_ replaces T_0_ in Equation (3), which is determined by the relative permittivity of the material and the Efros–Shklovskii localization length.

**Figure 5:**
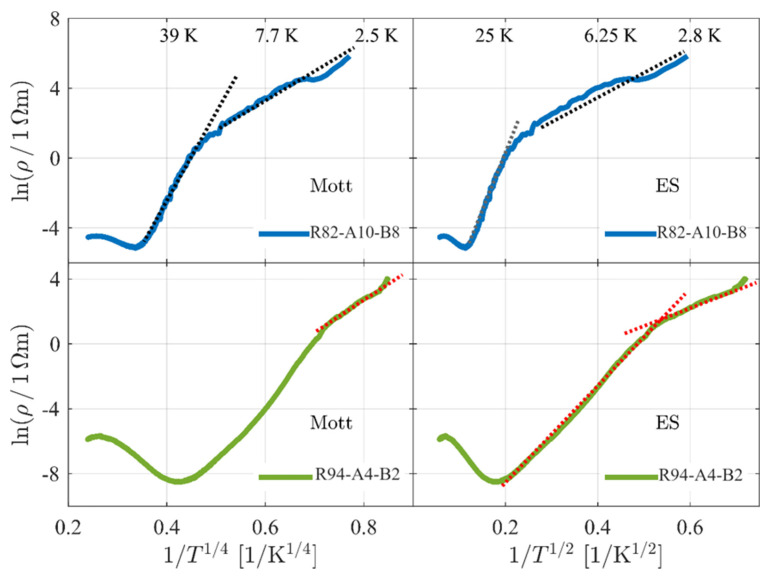
The representation of resistivity data with respect to temperature shows distinct regions attributed to Mott (ln(ρ) vs. $T^{-1/4}$ and Efros–Shklovskii (ES, ln(ρ) vs. $T^{-1/2}$ variable-range hopping mechanisms. The left panels correspond to Mott, while the right panels correspond to ES mechanisms; R82–A10–B8 is analyzed in the upper panels, and R94–A4–B2 in the lower panels. Blue and red dotted lines serve as guides for the eye and emphasize the linear parts of the curves. The gray line in the upper-right plot indicates linearity that does not represent a real physical mechanism.

The logarithmic-derivative analysis did not yield a robust single *p* exponent over a broad temperature range in any of the data. This indicates that the transport cannot be described by one pure VRH mechanism across the entire certain-temperature interval. Instead, the data presented in Figure 5 suggests the coexistence of transport regimes.

The stable-temperature regions for the validity of the Mott equation for R82–A10–B8 are found between 2–16 K and 27–60 K and for R94–A4–B2 in the 2–3.5 K range. For the Efros–Shklovskii equation, the fitting ranges are 2–13 K for R82–A10–B8 and 2–3.5 K and 4–25 K for R94–A4–B2. The seemingly linear Efros–Shklovskii range 27–60 K in R94–A4–B2 yields unphysically large T_ES_, so this linearity is not an indicator of real physical mechanism at these temperatures. All reported Mott-VRH and Efros–Shklovskii VRH fits satisfy the sliding-window stability check and high R^2^.

Generally, both the Mott VRH and the Efros–Shklovskii VRH mechanisms are identified at both ranges in R82–A10–B8 and at a lower temperature range in R94–A4–B2, while the second temperature range in R94–A4–B2 exhibits linearity only for Efros–Shklovskii VRH. Extracted T_0_ and T_ES_ parameters were used to check for the potential crossover from one mechanism to another in each sample, using the relation [36,37,38]:

(4) $$T_{cross}=\frac{T_{ES}^{2}}{T_{0}}$$

R82–A10–B8 data did not give a meaningful ratio, but R94–A4–B2 data gives 5 K for a possible crossover from Efros–Shklovskii VRH to Mott VRH. We note that there is also linearity in the Mott plot at the same low-temperature interval, indicating the coexistence of the two VRH mechanisms or the possible absence of the real physical mechanism in this range. Nevertheless, it is worth noting that the calculated 5 K corresponds to real temperature where the change in slope occurs in R94–A4–B2 (Figure 5, lower panels).

Observation of both the Mott and Efros–Shklovskii VRH supports the presence of localized carriers, such as electrons on Ti^3+^ ions [33,34,36,39]. From T_ES_, one can estimate the localization length ξ of the electron in the ES-VRH regime using the relation [36]:

(5) $$\xi =\frac{C_{ES}e^{2}}{k_{B}\epsilon_{0}\epsilon_{r}T_{ES}}$$

*C_ES_* ≈ 2.8 is the standard value for 3D ES-VRH, *e* is the unit charge, *k_B_* is the Boltzmann constant and *ϵ_r_* is the relative permittivity for TiO_2_. Using *ϵ_r_* = 35 for anatase and *ϵ_r_* = 90 for rutile [5,32], R82–A10–B8 yields localization lengths 4–11 nm in the 2–13 K range. R94–A4–B2 yields localization lengths 3–8 nm in the 2–3.5 K range and 0.6–1.5 nm in the 4–25 K range. All extracted VRH parameters are presented in Table 3.

A series of magnetoresistance measurements at several low temperatures were performed to further explore the dominant conduction mechanism. A standard 4-point contact method was used for measurements, with contacts on the sample established manually. This brought geometrical asymmetry into the measurements, and, therefore, symmetrization, using the equation

(6) $$\rho_{MR}\left(B\right)=\frac{\rho \left(B\right)+\rho (-B)}{2}$$

(6) was performed to obtain the magnetoresistance (MR) data:

(7) $$MR=\frac{\rho \left(B\right)-\rho (0)}{\rho (0)}$$

Positive magnetoresistance up to a maximum of 320% for R82–A10–B8 (at 2.1 K) and 550% for R94–A4–B2 (at 2.5 K) was observed at the lowest temperatures. With increasing temperature, positive magnetoresistance decreases to 25% at 6 K for R82–A10–B8 and to 15% at 10 K for R94–A4–B2. For R82–A10–B8, a weak negative MR (approximately −1%) is observed at 50 K, and at higher temperatures it is hardly noticeable. For R94–A4–B2, a weak positive MR is observed up to 40 K. At higher temperatures, MR becomes very weakly negative (−0.5%) up to ~100 K, after which it flattens out completely.

It is observed that at low temperatures (T < 15 K) and at low fields (B < 5.5 T), magnetoresistance has quadratic dependence on the magnetic field, so the data was analyzed with respect to a low-field Efros–Shklovskii VRH equation for magnetoresistance [30]:

(8) $$\ln\left(\frac{\rho \left(B\right)}{\rho \left(0\right)}\right)=t_{2}\frac{e^{2}{a_{H}^{*}}^{4}}{c^{2}\hbar^{2}}{\left(\frac{T_{ES}}{T}\right)}^{3/2}\cdot B^{2}=K(T)B^{2}$$

**Figure 6:**
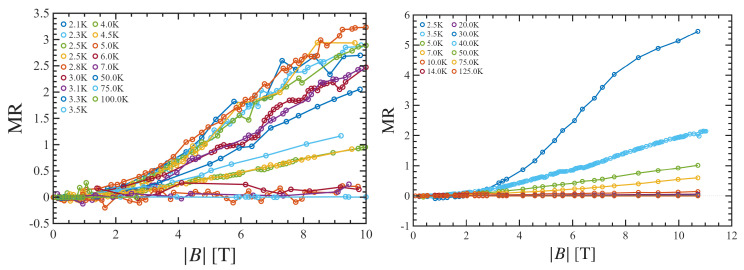
Symmetrized magnetoresistance data for selected temperatures: from 2.1 K to 100 K for R82–A10–B8 (**left**) and from 2.5 K to 125 K for R94–A4–B2 (**right**).

**Figure 7:**
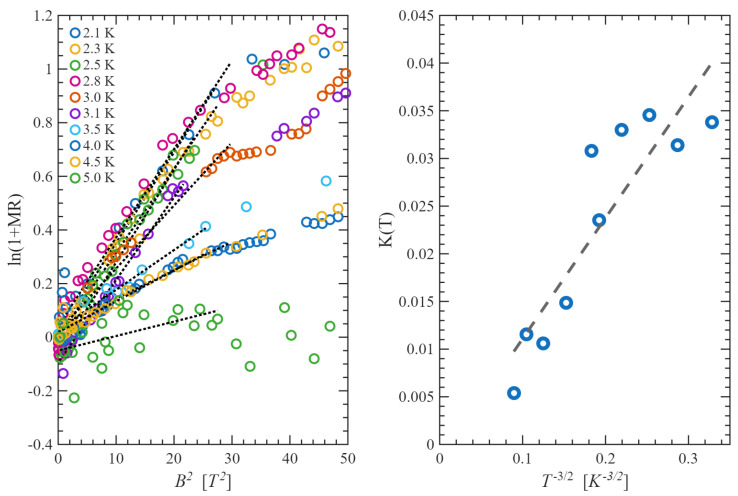
(**Left**): ln(1 + MR) as a function of $B^{2}$ for R82–A10–B8 for selected temperatures between 2.1 and 5 K. Dotted lines represent linear fits to the low-field data up to $B^{2}=30\;T^{2}$, yielding the slopes *K(T).* (**Right**): *K(T)* plotted as a function of T^−3/2^, showing the Efros–Shklovskii scaling.

$t_{2}\approx$ 0.0015 is a dimensionless numerical prefactor arising from the theoretical treatment of wavefunction shrinkage in a magnetic field, while $a_{H}^{*}$ is the effective Bohr radius without the presence of the magnetic field. The prefactor to $B^{2}$ is denoted as the temperature-dependent coefficient *K(T)*, which is proportional to $T^{-3/2}$. The linearity is presented in Figure 6 and Figure 7, with

(9) $$ln\left(\frac{\rho (B)}{\rho (0)}\right)\equiv ln(1+MR)$$

The observed $ln\left(\frac{\rho (B)}{\rho (0)}\right)\propto B^{2}$ scaling at low fields and the linear dependence of the extracted slopes to the temperature factor $K(T)∼T^{-3/2}$ confirm the Efros–Shklovskii variable-range hopping conduction mechanism at work in our samples in the low-temperature regime.

## 4. Discussion

Magnetic susceptibility measurements for both samples exhibit Curie–Weiss behavior, with low-temperature values comparable to those reported for rutile single crystals [19] (~1·10^−5^ cm^3^/mol at 4.2 K). Such relatively large susceptibility values may be associated with dilute Ti^3+^ ions. The overall positive magnetic susceptibility in R94–A4–B2 may indicate higher doping with oxygen vacancies in R94–A4–B2 than in R82–A10–B8. Unlike our diamagnetic result for R82–A10–B8, room-temperature susceptibilities of anatase crystals in the literature range from (0.5–1.6)·10^−6^ cm^3^/mol [17,19]. The room-temperature susceptibility of R94–A4–B2 (5.8·10^−6^ cm^3^/mol) is consistent with literature values for single-crystal rutile (5.35·10^−6^ cm^3^/mol) [17,19]. Although R94–A4–B2 exhibits a higher rutile fraction according to the preliminary SCXRD analysis, its weak residual paramagnetic response at 300 K suggests that the magnetic behavior is not governed solely by the phase composition. Even in such morphology, localized Ti^3+^-related states, oxygen-vacancy defects and interfacial regions may still contribute to weak paramagnetic signal superimposed on the dominant diamagnetic background. Curie–Weiss analysis yields small and negative Curie–Weiss temperatures, −9.1 K and −3.5 K, which may indicate weak antiferromagnetic interactions. In TiO_2_, these moments are usually associated with Ti^3+^ ions originating from oxygen vacancies [40,41] and possibly with trace paramagnetic impurities. In the literature, the reduction of oxygen ions from the regular TiO_2_ lattice is the main route for increasing the electron concentration in the sample. Even though the reduction was not performed on our samples, a certain amount can still be present in them, originating from the synthesis process. The extracted effective moments $\mu_{eff}\approx 0.1\mu_{B}$ are only 5% of the spin-only values expected for Ti^3+^ (1.73 $\mu_{B}$) [14], indicating a dilute concentration of localized magnetic moments (≈0.25%). We also measured and compared zero-field curves and field-susceptibility curves. For both samples, they show no significant splitting nor any maxima, which indicates magnetic reversibility and suggests that the observed magnetic response is intrinsic, governed by paramagnetic and diamagnetic contributions. This is also consistent with EDS results in Table 1, which show that the samples are without significant impurities that could affect the magnetism.

Moreover, both samples are black in color; the black coloration in TiO_2_ is typically associated with oxygen vacancies and Ti^3+^-related defect states [42,43]. Oxygen vacancies contribute with excess electrons which introduce mid-gap states that enable enhanced visible-light absorption [42]. In our sample, only 0.25% of localized magnetic moments behave as free spins. This implies that most vacancy-derived electrons may be either delocalized, paired antiferromagnetically, or magnetically quenched in polaron-like states, and are therefore silent in the susceptibility measurements. In the case of the first scenario, delocalized electrons would imply metallic-like electrical resistivity, which is not found in our samples at investigated lower temperatures. It may be present only at higher temperatures in the ρ(T) curves (Figure 3, >60 K for R82–A10–B8 and >20 K for R94–A4–B2). The second possible scenario, antiferromagnetic pairing, would result in a higher absolute value of Curie–Weiss temperatures than those that are reported here (Table 2). In contrast with the previous two options, the third option, polaronic behavior of charge carriers, has been confirmed in the literature for single crystals and well-controlled surfaces of TiO_2_ through spectroscopic and transport measurements. These studies reveal the formation and hopping of small polarons associated with Ti^3+^ centers and oxygen vacancies [39,44,45]. Polaron is a quasiparticle that consists of a self-trapped electron in a local Coulomb field created by a distorted lattice surrounding the electron. In TiO_2_, oxygen vacancy leaves two excess electrons, which can be located on a regular Ti^4+^ site, creating a Ti^3+^ polaron with surrounding lattice. Since the high polycrystallinity of our samples generates significant noise and a strong temperature-independent background, the polaronic-activated behavior could not be determined reliably from susceptibility measurements. It is therefore analyzed through presented resistivity and magnetoresistance measurements. Polaronic contributions to susceptibility are expected to be weak, and masked by the diamagnetic background, consistent with previous reports [7,11,12].

Resistivity vs. temperature data is presented in Figure 3. The difference between single-crystal values and R82–A10–B8 data is visible at low temperatures: R82–A10–B8 has two-orders-of magnitude higher resistivity value than single-crystal anatase [28] and two-orders lower resistivity value than single crystal rutile. The difference in low-temperature values nicely coincides with the proposed mixed-phase polycrystalline structure of R82–A10–B8. The low-temperature value for R94–A4–B2, mostly in rutile form, is two-and-a-half orders of magnitude lower than the value for rutile single crystal. This signifies the potential importance of anatase- and brookite-phase inclusions in the reduction of total resistivity of the polycrystal [46]. The difference between the decrease in resistivity with respect to the anatase- and brookite-structure part in the polycrystal may reflect the role of grain boundaries in the resistivity increase. Namely, the lower crystallinity and significance of grain boundaries in energy terms in R82–A10–B8 may compensate for the higher ratio of anatase, and possibly brookite, form in this sample.

The band gaps of rutile and anatase TiO_2_ are approximately 3.0 and 3.2 eV, respectively, and in both polymorphs defect–induced electronic states are reported to exist within the band gap, typically associated with oxygen vacancies and Ti^3+^ small polarons [34,47]. The proposed theoretical band-gap of the brookite structure is in the range 3.1–3.4 eV [16]. At low temperatures, these localized states dominate the electronic behavior, as charge carriers become trapped and transport occurs via hopping between in-gap states [15,32,33].

The very small low-temperature activation energy of 0.9 meV observed in both samples may be associated with carrier transport between proposed closely spaced localized Ti^3+^ states within an impurity band, which is often interpreted as nearest-neighbor hopping (NNH) conduction. In polycrystalline materials, defect states such as Ti^3+^ centers are known to accumulate at grain boundaries, which may act as preferential pathways for localized hopping transport [13]. However, this energy scale is significantly lower than the one expected for intrinsic small-polaron hopping in TiO_2_, which typically involves energies of several tens of meV [15,35]. Instead, it more likely reflects donor-related transport within shallow states that may be associated with Ti^3+^ centers. In this case, charge transport may happen via thermally assisted hopping within a narrow impurity band. However, the very low value of ~1 meV also suggests that, instead of the activated description, there may be a crossover to a different transport mechanism, such as variable-range hopping (VRH) at these lowest temperatures. This is consistent with the fact that shallow donor levels in rutile TiO_2_ are typically located several meV below the conduction band minimum [30,34].

At higher temperatures, larger activation energies are observed, indicating a transition toward more strongly localized transport regimes. In R82–A10–B8, the activation energy of ~20 meV found in the 30–55 K temperature range is similar to the values reported for small-polaron hopping in TiO_2_ or thermally activated transport involving deeper localized states [29,35]. This temperature interval also coincides with a change in the dominant conduction mechanism observed in the resistivity data. This may be associated with a crossover from low-temperature hopping between shallow localized states to thermally activated transport involving stronger electron–phonon interactions. A similar behavior has been reported for anatase TiO_2_, where carrier mobility above ~50 K is governed by scattering with optical phonons [29]. In R94–A4–B2, the intermediate activation energy of ~4 meV at the temperature interval of 5–30 K may suggest a transport in a regime of weak localization, possibly representing a crossover between impurity-band hopping and fully developed small-polaron conduction.

To further elucidate the low-temperature transport mechanism, the resistivity data were analyzed within the framework of the VRH mechanism. In disordered systems with localized electronic states, charge transport is expected to follow Mott VRH between energetically favorable localized states over variable distances, reflecting a finite density of states at the Fermi level. For R82–A10–B8, the Mott model provides stable fits in two distinct temperature intervals, 2–16 K and 27–60 K (Figure 5). These findings indicate that Mott-type hopping may contribute over a large enough and stable temperature range, although not as a single continuous regime. Both intervals strongly overlap with the temperature ranges that exibit linearity in the activated representation in R82–A10–B8 (Figure 4). The overlapping indicates that the proposed hopping transport in these regions cannot be described by a single mechanism, but rather reflects a coexistence of thermally activated hopping and VRH processes. In particular, the higher-temperature interval (27–60 K),associated with an activation energy of ~20 meV, may indicate small-polaron hopping or thermally activated transport involving deeper localized states. The lower-temperature interval (2–16 K) may suggest hopping within a proposed shallower impurity-band-like distribution of Ti^3+^ states. Within these same temperature ranges, the linearity in the Mott model suggests that carriers optimize both hopping distance and energy, characteristic of VRH conduction. This overlap, therefore, may indicate a gradual crossover between nearest-neighbor or polaron-assisted hopping and Mott-type VRH, governed by the evolving balance between thermal activation and disorder-induced localization.

In R94–A4–B2, by contrast, Mott VRH is limited to a narrow low-temperature interval of 2–3.5 K. This difference suggests that transport in R82–A10–B8 may be more strongly affected by structural and energetic inhomogeneity, which is possibly related to its mixed-phase, more pronounced, polycrystalline character. In such a heterogeneous system, it is plausible for multiple conduction mechanisms to coexist, due to the presence of numerous grain boundaries and microcrystalline regions. At lower temperatures, the transport in R82–A10–B8 may also be well described by Efros–Shklovskii (ES) VRH, which arises from the opening of the Coulomb gap due to electron–electron interactions. The Coulomb-gap-controlled hopping in a dilute localized electron system, such as TiO_2_, has been proposed theoretically by Shklovskii and Efros [36]. The ES model provides stable fits in the 2–13 K range. This range largely overlaps with the temperature interval where Mott VRH and activated behavior are also observed. The overlap suggests that multiple transport mechanisms coexist in this regime, reflecting a broad distribution of localized states.

In R94–A4–B2, ES-type linearity is observed in the low-temperature range, 2–3.5 K, where it coincides with Mott and activated linearities. It is also observed in an intermediate temperature range (4–25 K), which overlaps with the activated regime. This suggests that ES VRH may play a more dominant role in R94–A4–B2 over a wider temperature range, which may be in agreement with possible stronger carrier localization and enhanced Coulomb interactions. It can possibly be related to the suggested structurally more uniform nature of R94–A4–B2, which may be dominated by a central rutile crystallite. In this case, the single dominant transport mechanism within a given temperature interval may be favored. Also, the crossover temperature, estimated from Equation (4) to be ~5 K, is in good agreement with the experimentally observed change in the slope in the resistivity data (Figure 5, lower panels), but it should be treated with caution, since there is a simultaneous linearity in the ES representation in the 2–3.5 K interval.

Although an additional apparent linearity in the ES representation is observed at higher temperatures in R94–A4–B2, it yields unphysically large values of the characteristic temperature T_ES_. Too high T_ES_ indicates that this interval does not correspond to a physically meaningful ES transport regime.

The carrier localization parameter ξ, calculated from the ES fits using Expression (5), is higher in the low-temperature ES range (3–8 nm) for R94–A4–B2 than in the intermediate-temperature ES range (0.6–1.5 nm). This behavior may reflect the temperature-dependent hopping conditions in the presence of a Coulomb gap: at the lowest temperatures, charge transport may happen via long-distance hops between localized states that are closer in energy. This results in an effectively larger localization length. As the temperature increases, carriers may gain access to a broader range of energies, reducing the need for long-distance hops and leading to smaller effective hopping distances. Furthermore, at the lowest temperatures, R94–A4–B2 exhibits overall smaller ξ values compared to R82–A10–B8 (ξ~4–11 nm). This comparison indicates stronger carrier localization, consistent with a proposed more structurally uniform, rutile-dominated character.

**Figure 8:**
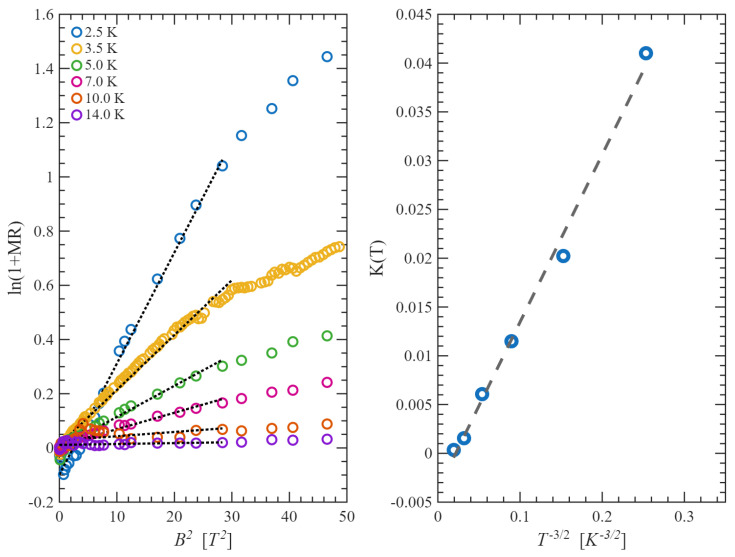
(**Left**): ln(1 + MR) as a function of $B^{2}$ for R94–A4–B2 for selected temperatures between 2.5 K and 14 K. Dotted lines represent linear fits to the low-field data up to $B^{2}=30\;T^{2}$, yielding the slopes *K*(*T*). (**Right**): *K*(*T*) plotted as a function of T^−3/2^, showing the Efros–Shklovskii scaling.

The observed low-field (<5.5 T) scaling $\ln\left(\frac{\rho \left(B\right)}{\rho \left(0\right)}\right)\sim B^{2}$, defined by Equation (8), and the linear dependence of the extracted slopes *K(T)* with respect to $T^{-3/2}$, $K\left(T\right)\sim T^{-3/2}$, is suggestive of Efros–Shklovskii variable-range hopping conduction in the low-temperature regime of both samples. The ES-type magnetoresistance is experimentally observed up to approximately 5 K in R82–A10–B8 (Figure 7) and up to 14 K in R94–A4–B2 (Figure 8). This behavior is compatible with the resistivity analysis (Figure 5, right panels), where ES VRH was proposed between 2–13 K for R82–A10–B8 and in two intervals, 2–3.5 K and 4–25 K, for R94–A4–B2. While the exact temperature limits differ, both approaches consistently indicate a broader stability of the ES regime in R94–A4–B2, compared to R82–A10–B8.

Within the ES framework, the applied magnetic field induces a shrinkage of the carrier wavefunction. This reduces the overlap between localized states, and consequently suppresses the hopping probability. Because of the presence of the Coulomb gap, the number of available low-energy states is strongly reduced. The reduction in states limits the number of accessible final states for hopping and increases the energetic cost of charge transport. This mechanism is proposed to be present in our samples at low temperatures (<5 K and <14 K) and at low magnetic fields (<5.5 T). This picture is further supported by the magnetic susceptibility results, which indicate only a small fraction of paramagnetic centers, which are possibly Ti^3+^ centers. This scenario would suggest that the majority of charge carriers are not free, but are instead trapped in localized or magnetically inactive states. They are participating in hopping conduction, but only a small fraction contribute to the Curie-like response. The proposed reduction in the wave-function at low temperatures (<5.5 K and <14 K) and in low magnetic fields (<5.5 T) raises the resistivity significantly. The localization of the electrons is presumably strong, and the concentration of the Ti^3+^ centers is considered to be low—there is no screening of the charge in the system.

In addition to the magnetoresistance scaling, the activation energies extracted from ρ(T,B) measurements (Figure 3, right panels) show a systematic increase with magnetic field in both samples. This behavior corroborates with the same proposed underlying mechanism of field-enhanced localization. As the magnetic field reduces the spatial extent of the electronic wavefunction, the overlap between neighboring localized states decreases. This effect leads to a reduced hopping probability and an effective increase in the activation energy. The comparable values of dE_a_/dB across different temperature intervals may indicate that this effect may be present both in the lowest-temperature ES regime and in the higher-temperature activated transport regime.

The strong positive magnetoresistance observed at low temperatures (<5 K and <14 K) and in low magnetic fields (<5.5 T) in our samples may therefore be the consequence of the interplay between Coulomb interactions and wavefunction shrinkage. In this scenario, the Coulomb gap suppresses the density of states near the Fermi level, while the applied magnetic field further reduces the spatial overlap of localized electronic states. These effects enhance localization and increase resistivity. This behavior would also be consistent with proposed charge carriers localized on Ti^3+^ centers and with the localization lengths obtained from the ES analysis (ξ~4–11 nm for R82–A10–B8 and ~0.6–8 nm for R94–A4–B2), which indicate stronger localization in R94–A4–B2. The absence of negative magnetoresistance at low temperatures, and the lack of a characteristic logarithmic field dependence, indicates the absence of weak localization as the dominant transport mechanism in the investigated temperature and field range.

Importantly, the magnetoresistance results corroborate the coexistence of transport mechanisms inferred from the resistivity analysis. Activated transport and Mott VRH are proposed to contribute over broad temperature ranges, but the ES mechanism is suggested to dominate at the lowest temperatures, where Coulomb interactions may become significant. The consistency between the inferred transport and magnetoresistance analyses may therefore suggest a broadly unified picture of charge transport. This transport may be governed by hopping between suggested localized Ti^3+^-related states in a disordered, polycrystalline environment. However, this description is not expected to remain universally valid over the entire temperature and magnetic-field range. In polycrystalline TiO_2_ systems, additional contributions may arise from grain-boundary-induced localization and intergrain hopping, particularly at higher magnetic fields and outside the low-temperature ES-VRH regime. Disorder-induced localization effects and crossover between Mott and Efros–Shklovskii VRH regimes may be difficult to distinguish at low temperatures [48]. Partial orbital magnetoresistance contributions at higher magnetic fields may also contribute to the deviations from the low-field quadratic dependence. The high-field magnetoresistance regime will be analyzed separately, to address possible orbital and interaction-driven effects beyond the low-field ES framework.

## 5. Conclusions

The combined magnetic, transport and magnetoresistance measurements consistently indicate that charge transport in studied TiO_2_ samples may be governed by defect-induced localized states. These states are suggested to be associated with Ti^3+^ centers. Magnetic susceptibility reveals only a small fraction of free paramagnetic spins, implying that most charge carriers are magnetically inactive and participate in hopping transport. The resistivity analysis demonstrates a coexistence of transport mechanisms at low temperatures, including nearest-neighbor hopping, Mott variable-range hopping and Efros–Shklovskii hopping. This interpretation is further supported by the observed positive magnetoresistance and its characteristic $B^{2}$ and $T^{-3/2}$ scaling at low temperatures and low fields. These results imply the dominant role of Coulomb interactions and wavefunction shrinkage in the Efros–Shklovskii regime. Overall, the results may point to a heterogenous, defect-controlled electronic structure. We suggest that a dilute population of localized Ti^3+^ polarons may coexist with spatially inhomogeneous conduction pathways. These pathways could be influenced by grain boundaries, giving rise to overlapping transport regimes across different low-temperature ranges. The presented findings highlight the role of disorder, phase composition, and Coulomb interactions in determining the low-temperature, low-magnetic-field electronic transport in mixed-phase TiO_2_ systems.

## Figures and Tables

**Table 1:** Atomic percentages (at.%) obtained from EDS analysis for R82–A10–B8 and R94–A4–B2. The values are used as qualitative indicators of the relative compositional differences and presumed structures of polycrystalline TiO_2_ samples. Carbon content presented in the table originates from mounting the samples with carbon tape, and should be excluded from consideration.

R82–A10–B8 [at.%]	R94–A4–B2 [at.%]
O|55.3	O|53.5
C|27.7	C|26.8
Ti|16.9	Ti|19.7
Al|0.1	

**Table 2:** Curie–Weiss fitting parameters and effective magnetic moments per $Ti^{3+}$ ion.

	R82–A10–B8	R94–A4–B2
Best temperature range for fitting	5−25 K	15 K−300 K
$\theta_{CW}\;\left[K\right]$	−9.1 K	−3.5 K
$C\;\left[\frac{cm^{3}K}{mol}\right]$	$1.3\cdot 10^{-3}$	$0.9\cdot 10^{-3}$
	$-3.6\cdot 10^{-5}$	$0.3\cdot 10^{-5}$
	0.102	0.083
Ti^3+^ per formula unit [%] *	0.4	0.2

* estimated assuming localized Ti^3+^ (S = ½, g = 2) centers.

**Table 3:** Characteristic transport parameters obtained from Mott and Efros–Shklovskii variable-range hopping fitting analyses for Samples 1 and 2, including the characteristic temperatures $T_{0}$ and $T_{ES}$, localization length in Efros Shklovskii regimes ξ, fitting-temperature ranges and corresponding coefficients of determination $R^{2}$.

Parameter	R82–A10–B8 $\mathrm{Temp}.\;\mathrm{Range}\;[K]|R^{2}$	R94–A4–B2 $\mathrm{Temp}.\;\mathrm{Range}\;[K]|R^{2}$
$T_{0}[K]$	5.23·10^4^ 2–16|0.98 1.01·10^7^ 27–60|0.99	1.57·10^5^ 2–3.5|1
$T_{S}[K]$	120 2–13|0.97	159 2–3.5|0.99 890 4–25|1
	4–11 2–13|-	3–8 2–3.5|- 0.6–1.5 4–25|-

## Data Availability

The raw data supporting the conclusions of this article will be made available by the authors on request.
